# A Bayesian approach to discrete multiple outcome network meta-analysis

**DOI:** 10.1371/journal.pone.0231876

**Published:** 2020-04-28

**Authors:** Rebecca Graziani, Sergio Venturini

**Affiliations:** 1 Department of Social and Political Sciences, Bocconi University, Milan, Italy; 2 Dondena Centre for Research on Social Dynamics and Public Policy, Bocconi University, Milan, Italy; 3 Bocconi Institute for Data Science and Analytics, Bocconi University, Milan, Italy; 4 Dipartimento di Management, Università degli Studi di Torino, Torino, Italy; 5 Centre for Research on Health and Social Care Management (CeRGAS), SDA Bocconi School of Management, Milan, Italy; Cleveland Clinic Lerner Research Institute, UNITED STATES

## Abstract

In this paper we suggest a new Bayesian approach to network meta-analysis for the case of discrete multiple outcomes. The joint distribution of the discrete outcomes is modeled through a Gaussian copula with binomial marginals. The remaining elements of the hierarchial random effects model are specified in a standard way, with the logit of the success probabilities given by the sum of a baseline log-odds and random effects comparing the log-odds of each treatment against the reference and having a Gaussian distribution centered at the vector of pooled effects. An adaptive Markov Chain Monte Carlo algorithm is devised for running posterior inference. The model is applied to two datasets from Cochrane reviews, already analysed in two papers so to assess and compare its performance. We implemented the model in a freely available R package called netcopula.

## Introduction

In the last decades, as the need of evidence based techniques in medical research and clinical practice has been more and more recognized, the use of meta-analysis, introduced with a high level of debate, has become widespread. Nowadays, areas of application of meta-analysis extend beyond medicine and health, being widely used in both natural and social sciences. See [1] for a critical review of the main methodological developments in meta-analysis. Traditionally, meta-analytic techniques make it possible to summarize evidence provided by several studies comparing the same treatments and considering in general one outcome at time. The basic methods combine study-specific treatment effect estimates under a fixed effect or a random effect model (see [2] and [3]). Study specific covariates and individual patients data can be incorporated as well (see for IPD meta-analysis among others [4–6] and [7]). Bayesian methods are widely used, making it possible to allow for all parameter uncertainty in the model, to include all relevant information and to extend the models to accommodate more complex scenarios. Advantages of the Bayesian approach are discussed and reviewed in several papers and books, see among others [8] and [9].

Meta-analytic techniques have been developed in the recent years along several directions. In the present work, we focus on one of the most recent development, multiple outcomes network meta-analysis. Network meta-analysis in the past few years has become increasingly popular and its advantages and disadvantages are discussed in several published articles, see among others [10–14] and [15]. In particular [16] provide a complete and detailed review of network meta-analysis techniques. Network meta-analysis makes it possible to combine both direct and indirect evidence provided by different studies with respect to different treatments. The main challenge is to use the totality of trial evidence to determine an internally consistent set of estimated treatment effects between all treatments, while respecting randomization. As long as the included trials and treatments form a connected network, network meta-analysis allows to borrow strength across treatments in the estimation of relative effect sizes. Full-fledged ranking of all considered treatments can be also obtained. Several methods for running univariate network meta-analysis have been proposed and coded in statistical packages. However, there are relatively few attempts to extend these methods to the multivariate setting.

The need of multiple outcomes network meta-analytic techniques stems from the fact that often studies report several outcomes, that in general are correlated. Such correlation arises when several outcomes are measured on the same participants, when one event is nested in another (as in the case of disease survival nested in total survival) or when outcomes are measured repeatedly on the same participants. The outcomes correlation entails a correlation of the treatments effect, which is clearly neglected if separate univariate network meta-analyses are run for each outcome. Multiple outcomes network meta-analysis makes it possible to account for such within-study correlation of the treatment effects so to simultaneously borrow strength across treatments and outcomes. In this way, more studies contribute towards each outcome and treatment comparison. Indeed, summary results for each outcome depend on correlated results from other outcomes, and summary results for each treatment comparison incorporate indirect evidence from related treatment comparisons, in addition to any direct evidence.

Multiple outcomes network meta-analysis faces the same issues that multiple outcomes meta-analysis addresses, but in a more complicated setting, see [17] and [18] for an overview of advantages and disadvantages of multiple outcomes techniques. We can identify two main challenges. The first stems from the fact that while studies report estimates and standard errors of the treatments relative effect for each outcome, rarely the corresponding covariance matrix is provided. In multiple outcomes meta-analysis, a common choice is to assume that the within-study covariance is known and to focus on the estimation of the between-study covariance matrix. In a frequentist approach the entries of such matrix are estimated resorting to maximum likelihood techniques as in [19] or restricted maximum likelihood techniques as in [20]. Method of moments techniques are used as well, [21] and [22] suggest a multivariate generalization of the DerSimonian and Laird’s methodology. [23] suggest a structural equation modeling approach (see [24] for a review on the use of such approach in meta-analysis). The within-study correlation coefficients are then imputed on the basis of individual patients data, when available for similar studies. When individual patients data are not available, plausible values can be assumed on the basis of clinical considerations as in [25]. As well empirical correlations can be used as in [20]. [26] use delta methods to approximate the within-study correlation on the basis of information on the outcomes correlation. [27] suggest a Bayesian approach to multiple outcome meta-analysis, where the within study variances are assumed to be known and simplifying assumptions are made on the correlations so to reduce the number of parameters. Noninformative priors are assigned to such correlations. [28] use external sources of information to construct informative priors for both within-study correlations and the between-study covariance matrix.

The second issue to be addressed in a multivariate generalization of network meta-analytic techniques comes from the fact that not all studies report results on all the considered outcomes. In multiple outcomes meta-analysis, the missing outcomes issue is addressed relying on traditional imputation and data augmentation techniques both in a frequentist and a Bayesian approach ([29, 30]). In a Bayesian approach [26] express the likelihood as a product of marginal distributions over reported outcomes following the approach suggested by Glester and Olkin in [31]. Such techniques make it possible to borrow strength across outcomes and this, as pointed out by [32], reduces the impact of a selective non reporting of the outcomes on the pooled treatment effect estimates.

In this work we adopt a contrast-based perspective to estimate the treatment effects in a network meta-analysis, see [33, 34]. Contrast-based models currently represent the most popular methodology in the network meta-analysis literature. However, another approach, called arm-based, has also been recently advanced (see [35–38]). In contrast-based models, a baseline treatment is defined for each study and the focus of the analysis is on the estimation of the relative treatment effects (for example using log odds ratios, or another suitable metric). In this context, the baseline effects are treated as nuisance parameters and they are usually modeled with noninformative prior distributions. This implies that absolute treatment effects cannot be directly obtained unless a reference treatment absolute effect is first estimated using information that are external to the model see among others [39]. On the contrary, arm-based models aim to model the absolute effect of each treatment in a study (for example using the log odds) and the relative treatment effects are then constructed from the arm estimates. Contrast-based models are usually advocated as more theoretically grounded compared to the arm-based approach because the latter discards the randomization structure of the evidence. Moreover, arm-based models are more likely to provide biased estimates of relative treatment effects with increased posterior variances, and they often show a slower convergence especially when some treatments are only included in few studies. On the contrary, arm-based models are more advantageous because they can also incorporate the information provided by single-arm studies. For more details on the pros and cons of the two approaches see [40] and the discussion rejoinder by [41], while for a more technical comparison between arm-based and contrast-based models for network meta-analysis we suggest the recent work by [42].

In this paper we present a new approach to network meta-analysis in the case of discrete multiple outcomes. The model we suggest is a Bayesian hierarchical random effects model that is based on a Gaussian copula likelihood, which allows to incorporate the estimation of within-study variances and correlations. Our approach draws on and generalizes the method suggested by [43] based on a Clayton copula model. However the switch from a Clayton copula model to a Gaussian copula model is not straightforward due to the implications in terms of computational problems to be addressed. Indeed in the case of Clayton copula model the correlation between outcomes is modeled through a single univariate parameter, while in the case of a Gaussian copula model the association is modeled through a correlation matrix. Posterior inferences are based on a latent variables adaptive Markov Chain Monte Carlo algorithm, that draws on the suggestions by [44] and [45] for copula regression models and by [46] for the simulation of correlation matrices. The uncertainty due to the missingness problem is addressed and accounted for through a posterior based imputation of missing outcomes at each stage of the algorithm. All codes, data and examples are available in a R package called netcopula, that can be freely downloaded from the following public repository https://github.com/sergioventurini/netcopula.

The set-up of the paper is as follows. Section 2 provides a description of the suggested model and of the MCMC algorithm devised for running posterior inferences. In Section 3 two applications of the model are provided with a comparison with other two different approaches. Section 4 provides a discussion with final conclusions.

## Method

### Introduction

Copula models have become widely used in all applied fields since they make it possible to split the specification of a multivariate model into two parts: the marginal distributions on one side and the dependence structure on the other side. In this way, any univariate distribution can be used for modelling the marginal behavior of the considered variables, which can be discrete and continuous. Moreover, marginal distributions belonging to different families can be selected, ensuring a higher flexibility with respect to a traditional modeling with multivariate distributions. The dependency across the variables is then modelled through a copula function that “glues together” the marginal distributions.

In the following, we briefly review the copula based approach for the case of two variables, *Y*_1_ and *Y*_2_, with marginal cumulative distributions *F*_1_ and *F*_2_ respectively. We want to obtain a bivariate distribution for the vector (*Y*_1_, *Y*_2_) having these two margins. Sklar ([47]) proved that we can always find a function *C* such that

$$\begin{array}{c} F\left(Y_{1}=y_{1},Y_{2}=y_{2}\right)=C\left(F_{1}\left(y_{1}\right),F_{2}\left(y_{2}\right)\right) \end{array}$$ (1)

where *C*(*y*_1_, *y*_2_) is the joint distribution function for a pair of bivariate uniform random variables. Sklar called *C* copula function and showed three relevant properties (see [48] for a detailed introduction to copula models). The distribution in Eq (1) is constructed from the marginal distributions *F*_1_ and *F*_2_, while the role of the copula function is to determine the dependence between *Y*_1_ and *Y*_2_. If the marginal distributions are continuous, differentiating Eq (1) gives the joint density

$$\begin{array}{c} f\left(y_{1},y_{2}\right)=c\left(F_{1}\left(y_{1}\right),F_{2}\left(y_{2}\right)\right)f_{1}\left(y\right)f_{2}\left(y\right) \end{array}$$ (2)

where *c*(*F*_1_(*y*_1_), *F*_2_(*y*_2_)) is the copula density. Eq (2) shows that the copula density controls the level of dependence between *Y*_1_ and *Y*_2_. The copula function does not determine the distribution of the margins. It merely determines the dependence between the two random variables. There are many copula functions, one of the most popular is the Gaussian copula. In the continuous case a useful way to think at the copula method is that, based on the probability integral transformation on each margin, the original variables are each transformed into uniform random variables *U*_*j*_ = *F*_*j*_(*Y*_*j*_). Indeed no matter is the marginal distribution *F*_*j*_, *U*_*j*_ has a uniform distribution and the dependency between the original variables carries through to the transformed uniform distributions. In this way, assuming a copula model as in Eq (1) for the pair (*Y*_1_, *Y*_2_) reduces to considering the following model

$$\begin{array}{cc} Y_{j} & =F_{j}^{-1}\left(U_{j}\right)\;j=1,2\\ \left(U_{1},U_{2}\right) & ∼C(u_{1},u_{2}) \end{array}$$

In the case of discrete marginal distributions, the marginal are steps function, we define *U*_*j*_ as associated with the variable *Y*_*j*_ through the following inequality

$$\begin{array}{c} F_{j}\left(Y_{j}-1\right)<U_{j}<F_{j}\left(Y_{j}\right) \end{array}$$

Nevertheless this still ensures *Y*_*j*_ is uniformly distributed in the interval (0, 1). The method can be easily generalized to the case of more than two variables.

### Model specification

We consider a sample of *n* multi-arm randomized trials and let ***y***_*ik*_ denote the vector of the number of times each of *M* outcomes is observed in study *i* for treatment *k*, that is ***y***_*ik*_ = (*y*_*ik*1_, …, *y*_*ikM*_)^⊤^, $k\in T_{i}=\{1,\ldots ,a_{i}\}$, where $k\in T_{i}$ is the set of treatments compared in study *i*, with the treatment labelled as 1 representing the control (i.e. the baseline) treatment in study *i* whose efficacy is compared with that of the remaining (*a*_*i*_ − 1) treatments. Note that the term “treatment 1” may refer to distinct treatments in the different studies. We suggest to model ***y***_*ik*_ as the realization of a multivariate discrete random variable ***Y***_*ik*_, *i* = 1, …, *n* and $k\in T_{i}$, with distribution built from a Gaussian copula with binomial margins. We assume that for each study *i*, each arm *k* and each outcome *m*

$$\begin{array}{c} y_{ikm}=F_{ikm}^{-1}\left(\Phi \left(x_{ikm}\right)\;|\;n_{ik},p_{ikm}\right) \end{array}$$

where $F_{ikm}^{-1}\left(\cdot \;|\;n_{ik},p_{ikm}\right)$ is the inverse cumulative distribution function of a binomial random variable with parameters *n*_*ik*_ and *p*_*ikm*_, *n*_*ik*_ is the number of patients randomized to arm *k* in study *i*, *p*_*ikm*_ the treatment-specific probability of an outcome of type *m* in study *i* and *x*_*ikm*_ is the *m*-th component of vector ***x***_*ik*_, that is the realization of a random vector having a multivariate Gaussian distribution with a arm-specific correlation matrix, **Γ**_*k*_. As previously emphasized, *F*_*ikm*_ as the cumulative distribution function of a discrete random variable is a step function, therefore its inverse is a many-to-one function. This indeed complicates the calculations as compared to the continuous case (see [49]). The variables *x*_*ikm*_ are latent, not observed variables to be associated with each *y*_*ikm*_. The logistic transformations of the treatment-specific probabilities $\mathrm{logit}\left(p_{ikm}\right)=\theta_{ikm}$ are modelled for $k\in T_{i}=\{1,\ldots ,a_{i}\}$, as follows

$$\begin{array}{c} (\begin{array}{c} \theta_{ik1}\\ \vdots \\ \theta_{ikM} \end{array})=\{\begin{array}{ccc} \left(\begin{array}{c} \mu_{i1}\\ \vdots \\ \mu_{iM} \end{array}\right) & & \text{if}\;k=1\\ \left(\begin{array}{c} \mu_{i1}+\delta_{ik1}\\ \vdots \\ \mu_{iM}+\delta_{ikM} \end{array}\right) & & \text{if}\;k\in T_{i}^{-}=\left\{2,\ldots ,a_{i}\right\} \end{array} \end{array}$$

where, ***μ***_*i*_ = (*μ*_*i*1_, …, *μ*_*iM*_)^⊤^ denotes the vector of study-specific baseline effects and ***δ***_*ik*_ = (*δ*_*ik*1_, …, *δ*_*ikM*_)^⊤^ indicates the trial-specific log-odds ratios of treatment $k\in T_{i}^{-}$ relative to the baseline treatment (i.e. treatment 1) in study *i*.

Finally, we assume that the random effects have a multivariate normal distribution

$$\begin{array}{c} \left(\begin{array}{c} \delta_{i2}\\ \vdots \\ \delta_{ij}\\ \vdots \\ \delta_{ia_{i}} \end{array})∼N_{M\times (a_{i}-1)}((\begin{array}{c} d_{t_{i1},t_{i2}}\\ \vdots \\ d_{t_{i1},t_{ij}}\\ \vdots \\ d_{t_{i1},t_{ia_{i}}} \end{array}),\Sigma \right) \end{array}$$ (3)

where *t*_*i*1_ denotes the baseline treatment in study *i*, *t*_*ij*_ is the *j*-th treatment compared in study *i* and ***d***_*j*,*k*_ = (*d*_*j*,*k*,1_, …, *d*_*j*,*k*,*M*_)^⊤^ represents the vector of pooled effects (across trials) of treatment *k* relative to treatment *j*. The ***d***_*j*,*k*_ are usually the main quantities of interest in a meta-analysis. The consistency equations

$$\begin{array}{c} d_{t_{i1},t_{ij},m}=d_{r,t_{ij},m}-d_{r,t_{i1},m} \end{array}$$ (4)

where *r* identifies a treatment chosen as reference, ensure that the correct treatment comparison is used in the network meta-analysis (see [33] and [50]). Note that to guarantee consistency, it is also required that *d*_*r*,*r*,1_ = ⋯ = *d*_*r*,*r*, *M*_ = 0.

The matrix **Σ** in Eq (3) contains the variances of the random effect *δ*_*i*,*k*, *j*_ for each treatment *k* ∈ 2, …, *a*_*i*_ and each outcome *m* = 1…, *M*, and all possible covariances between any two random effects. As the pooled treatment effects, it is common to all studies and for this reason commonly referred to as a matrix that defines the between-study covariance structure, in opposition to the **Γ**_*i*_ that models the within-study correlation structure across outcomes. To keep the number of parameters manageable and to allow identifiability of **Σ**, we follow [51]and make the following simplifying assumption

$$\begin{array}{c} \Sigma =(\begin{array}{cccc} \Sigma_{M} & \frac{1}{2}\Sigma_{M} & \cdots & \frac{1}{2}\Sigma_{M}\\ & \Sigma_{M} & \cdots & \frac{1}{2}\Sigma_{M}\\ & & \ddots & \vdots \\ & & & \Sigma_{M} \end{array}) \end{array}$$

where

$$\begin{array}{c} \Sigma_{M}=(\begin{array}{ccc} \sigma_{1}^{2} & \cdots & \rho^{1M}\sigma_{1}\sigma_{M}\\ & \ddots & \vdots \\ & & \sigma_{M}^{2} \end{array}), \end{array}$$

describes the common between-study covariance structure. In this way, we assume that the variances and covariances of the random effects for each treatment comparison are the same and differ only with respect to the considered outcome. Moreover, such homogeneity assumption along with the structural relationships between the ***d***_*j*,*k*_ within trial *i* imply that the between-arm correlations are assumed to be all equal to 0.5 (see [52]). These correlations between the treatment differences come from the fact that all differences are taken relative to the same control arm, that is, they depend on the same trial baseline effect ***μ***_*i*_.

### Priors choice

As for the prior assignment, proper priors are selected and we specify them in the application so to be vague. The study-specific baseline effects ***μ***_*i*_ = (*μ*_*i*1_, …, *μ*_*iM*_) are assumed to be independent and distributed according to a normal distribution with mean zero and variance $\sigma_{\mu }^{2}$. As well the pooled (across trial effects) ***d***_*r*,*q*_ = (*d*_*r*,*q*,1_, …, *d*_*r*,*q*, *M*_) are assumed to be independent and identically distributed according to a normal distribution with mean zero and variance $\sigma_{d}^{2}$.

As for the matrix **Σ**_*M*_, it has been shown in the literature that a standard conjugate Wishart prior is overly influential on the corresponding posterior distribution (see among others [26, 53] and [54]). Moreover, explicitly representing an informative prior distribution for a covariance matrix is difficult. We therefore follow an alternative strategy by adopting a log-Cholesky parameterization (see [55]) for the precision matrix $\Sigma_{M}^{-1}$. More specifically, we define $\Sigma_{M}^{-1}=R^{⊤}R$, with ***R*** = {*r*_*m*,*p*_} being an upper triangular Cholesky factor, with *p* = 1, …, *M*, *m* ≤ *p*. To guarantee that the Cholesky factorization is unique, one has to require the diagonal elements of ***R*** to be positive. To avoid constrained estimation, we use the logarithms of the diagonal elements of ***R***. Hence, the covariance matrix is parameterized in terms of the parameter vector

$$\begin{array}{c} \beta =(\log r_{1,1},r_{1,2},\log r_{2,2},r_{1,3},r_{2,3},\log r_{3,3},\ldots ,\log r_{M,M}) \end{array}$$ (5)

Finally, we assume that the components of ***β***, *β* *_ℓ_* with *ℓ* = 1, …, *M*(*M*+ 1)/2, are a priori independent and all distributed according to a normal distribution with zero mean and variance $\sigma_{\ell }^{2}$.

The correlation matrices **Γ**_*q*_, with *q* ∈ *T* are assumed to be independent and uniformly distributed on the space of all correlation matrices.

### Posterior computations

**Fig 1:**
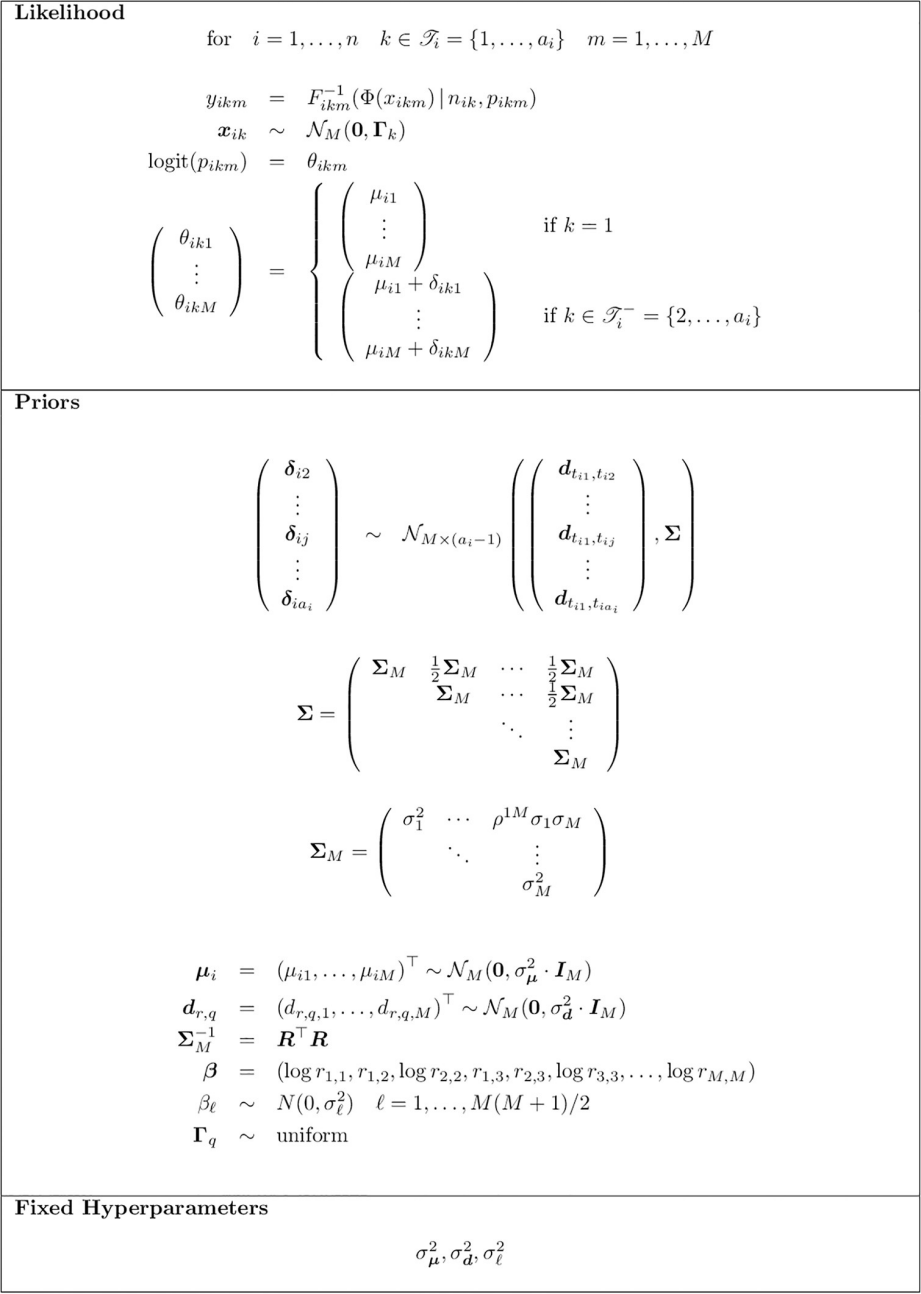
The multiple outcomes network meta-analysis model. The Figure depicts the elements of the suggested hierachical model.

Fig 1 summarizes the formulation of our hierarchical model. Since the joint posterior of the model parameters cannot be obtained in closed form, we devise a latent variables and adaptive Markov Chain Monte Carlo (MCMC) algorithm for the posterior inference. At each iteration, for each unit *i* (i.e. each study), two sets of latent variables are introduced: the random effects $\left(\delta_{i2},\delta_{i3},\ldots ,\delta_{ia_{i}}\right)$ and the latent vectors related to the specification of the copula model, ***x***_*ik*_, *i* = 1, …, *N*. Drawing on [45] and [44], we suggest to jointly update all latent variables and the baseline vectors *μ*_*i*_. This is the most delicate step of the algorithm. An adaptive metropolis step is as well foreseen for the updates of the random effects and the baseline log-odds.

The updates of the copula parameters **Γ**_*q*_ are obtained by applying the two-stage parameter expanded reparameterization and Metropolis-Hastings (PX-RPMH) algorithm for simulating a correlation matrix proposed by [46]. Finally, the full conditionals for ***d*** and **Σ**_*M*_ are obtained from standard results for Bayesian analysis of multiple regression models, an adaptive metropolis step is used for their simulation.

It is worth emphasizing here that the algorithm also allows for the possibility that the outcomes are reported differently in the studies. In this situation, a simple strategy one can implement consists in analyzing only the subset of events reported by all studies. Even if this suggestion allows to bypass the problem, the risk is that a considerable amount of data may be discarded. In our approach missing data are imputed at each iteration of the algorithm.

A detailed description of devised MCMC algorithm is provided in the Appendix.

## Results and discussion

We apply the suggested model to two datasets from two Cochrane reviews. The two datasets have been analysed based on two different models, so that the performance of our model can be assessed and compared against two different approaches. In both cases we assume that the studies share the same Gaussian copula correlation matrix **Γ** and diffuse priors are chosen for all parameters by setting in particular $\sigma_{\mu }^{2}$ and $\sigma_{d}^{2}$ equal to 10^3^ and $\sigma_{\ell }^{2}$ equal to 10^1/8^. In both cases not all outcomes are investigated in all studies. Missing outcomes value *y*_*ikm*_ are imputed at each iteration of the algorithm, by imputing the corresponding latent variable value *x*_*ikm*_, from the model predictive distribution. The results come from a long run of the devised algorithm with 350000 iterations of which 300000 are discarded as burn-in. In both cases the convergence of the algorithm is assessed through the R coda package. Both examples can be reproduced since the corresponding scripts are provided as demos.

### Home safety

**Fig 2:**
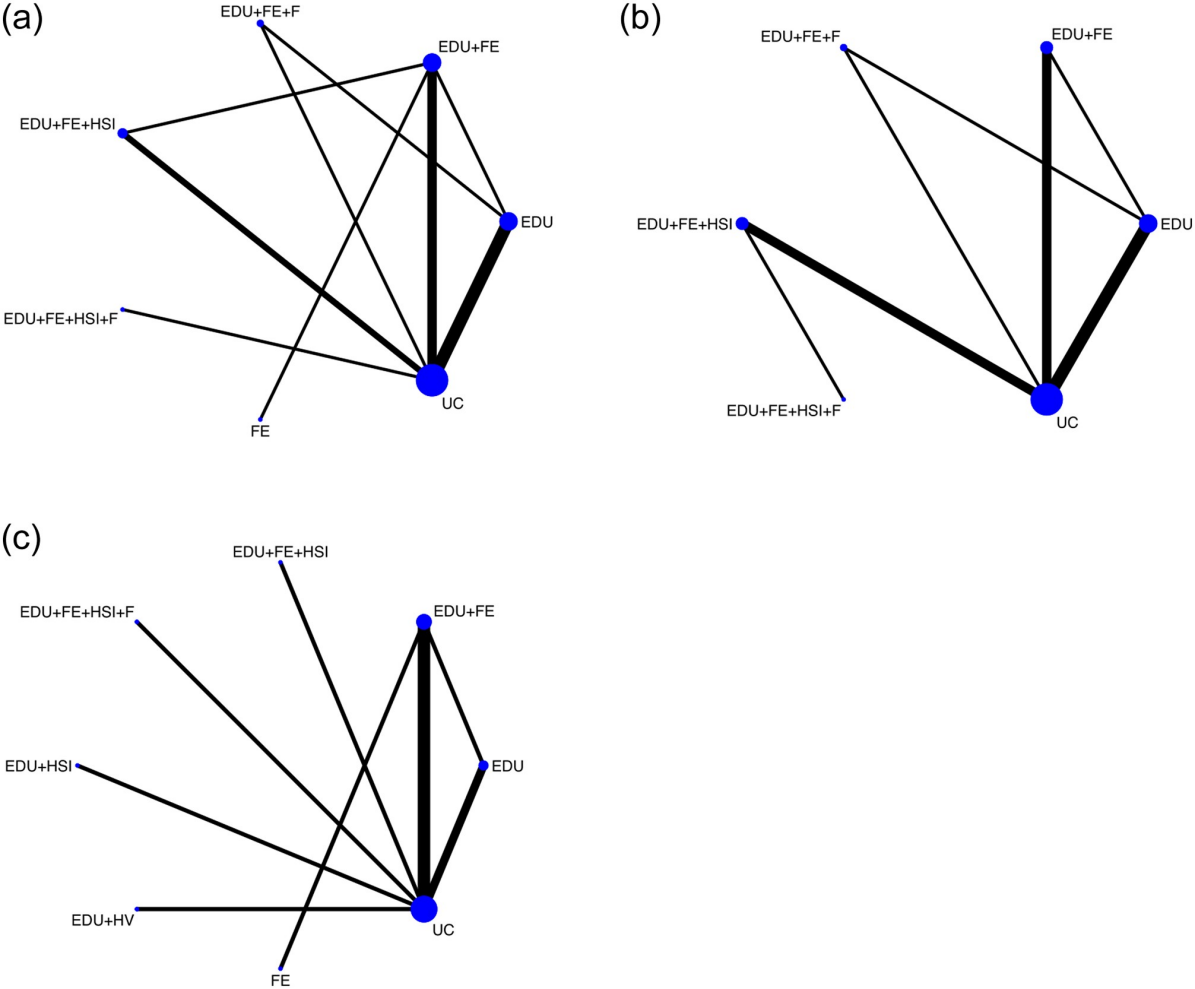
Home safety: The network structure. The figure shows the network structure: A: Medicines Safe storage, B: Household Products Safe Storage and C: Poison Center Number Possession. The thicker the lines the higher the number of studies reporting results on the considered outcome.

In the first example we consider the data from a subset of a Cochrane review of safety education and provision of safety equipment for injury prevention, see [56] for a description of the methods. The focus is on the evidence relating to the prevention of poisoning injuries. Data come from twenty-two studies on three outcomes are recorded: Medicines Safe storage, Household Products Safe Storage and Poison Center Number Possession. Nine treatments are considered: Usual Care (UC), Education (EDU), Education + Provision of free/low cost equipment (EDU+FE), Education + Provision of free/low cost equipment + Fitting of Equipment (EDU+FE+F), Education + Home Safety Inspection (EDU+HSI), Education + Provision of free/low cost equipment + Home Safety Inspection Fitting of Equipment (EDU+FE+HSI+F), Education + Home Visit (EDU + HV), Provision of Free/Low Cost Equipment (FE). Overall there are three studies considering all three outcomes, nineteen studies considering two outcomes. All studies but one are two arms. Fig 2 depicts the network graph for the three outcomes.

**Fig 3:**
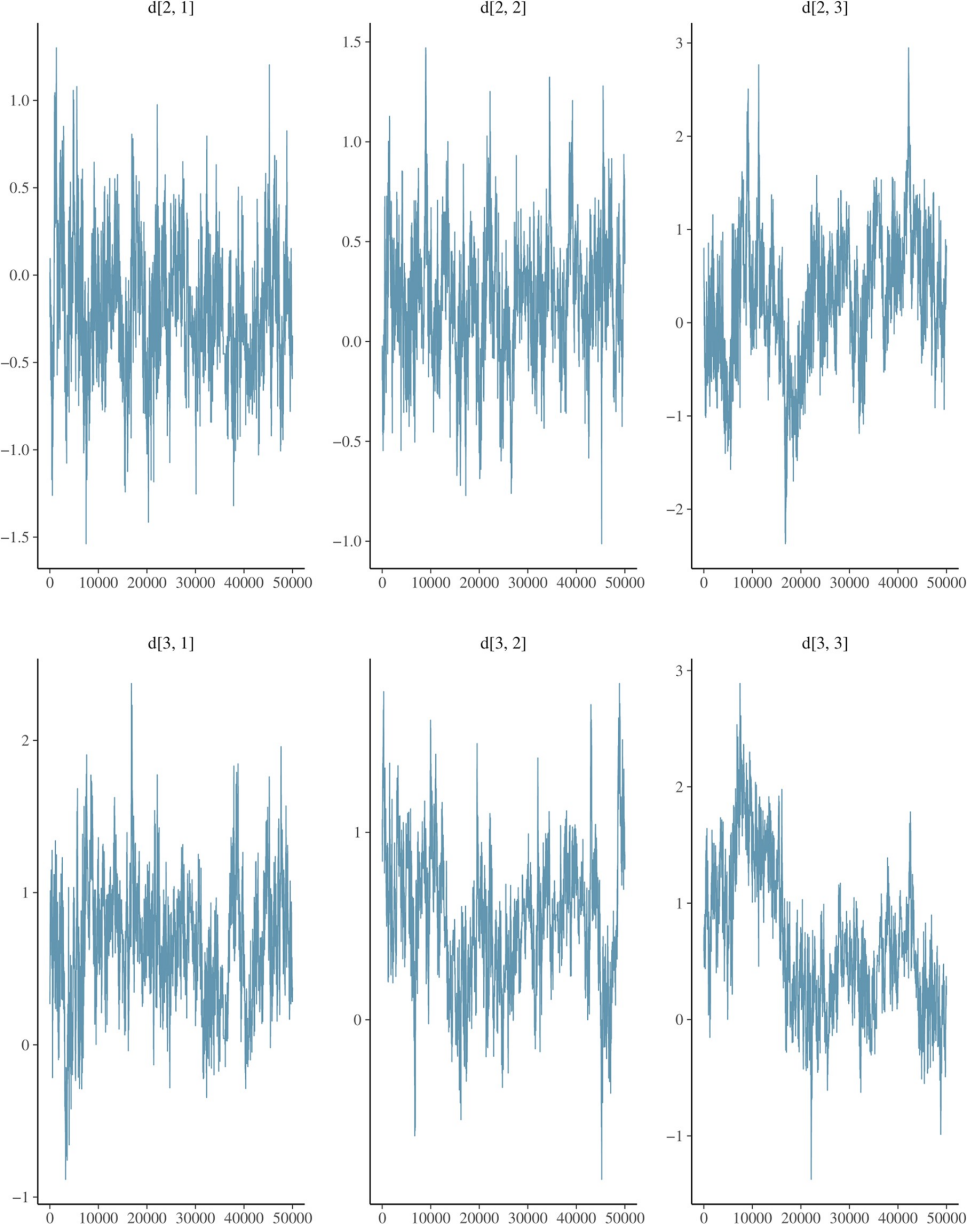
Home safety: Trace plots. The figure shows the trace plots of the pooled treatments effect against the baseline treatment Usual Care, after discarding the burnin.

[51] analyse the same dataset, based on a different approach. Indeed, [51] model the outcomes log odds ratio so that a continuous, Gaussian, multivariate distribution can be used as likelihood. Moreover, in [51] the within-study covariances are taken as known, as they are estimated from the data. In our approach, such matrices are assumed to be unknown so that the model provides as well an estimate of them. The analysis can be replicated by running the script example_homesafety.R to be found in the folder demo of the netcopula package. Fig 3 displays the trace plots of the pooled treatment effects against the baseline Usual Care.

Tables 1 to 3 display median estimates along with the highest posterior density (HPD) credibility intervals for the pooled effects estimated according to our model and according to [51], referred to as Model 3 in the original paper. Usual care is taken as baseline effect. In all cases our estimates show a smaller variability, in particular in the estimation of the pooled effect of Education + Home Visit versus Usual Care, of Education + Home Safety Inspection versus Usual Care and of Provision of Free/Low Cost Equipment versus Usual Care. The EDU+HV and EDU+HSI are indeed considered only for one outcome and the effect directly compared against Usual Care. The FE treatment is considered for two outcomes and only indirectly compared against Usual Care. The smaller uncertainty of the median estimates for such treatments shows that our model succeeds in borrowing strength across treatments and outcomes as foreseen. There are differences in the estimates of the pooled-effects for some treatment comparisons. In almost all cases our estimates belong to the corresponding credibility intervals in [51]. A simulation study (not reported here but available as a further demo in the netcopula R package) shows that, for different settings of the true parameters value, our model is able to recover the true values of the pooled effects.

**Table 1:** Safe storage of medicines: Pooled effects posterior median and HPD 95% credibility intervals.

treatments	Copula MONMA			Achana et al. MONMA (model 3)		
	median	2.50%	97.50%	median	2.50%	97.50%
EDU-UC	1.81	1.16	3.04	1.32	0.71	2.16
EDU+FE-UC	1.24	0.76	2.14	2.11	1.08	3.94
EDU+FE+HS-UC	1.16	0.82	1.39	1.93	1.06	3.94
EDU+FE+F-UC	1.94	1.02	2.99	1.27	0.68	2.43
EDU+HIS-UC	0.97	0.64	1.60	0.66	0.06	7.09
EDU+FE+HIS+F-UC	0.96	0.51	1.91	2.09	1.13	4.27
EDU+HV-UC	1.47	1.05	2.16	1.42	0.09	14.49
FE+UC	0.64	0.37	1.02	1.75	0.47	5.67

**Table 2:** Safe storage of other household products: Pooled effects median and HPD 95% credibility intervals.

treatments	Copula MONMA			Achana et al. MONMA (model 3)		
	median	2.50%	97.50%	median	2.50%	97.50%
EDU-UC	1.2	1.00	1.66	1.32	0.78	2.15
EDU+FE-UC	0.97	0.7	1.24	2.13	1.15	3.91
EDU+FE+HS-UC	1.45	0.94	2.32	1.95	1.12	3.93
EDU+FE+F-UC	1.3	0.98	1.67	1.26	0.67	2.59
EDU+HIS-UC	1.23	0.79	1.81	0.64	0.05	7.97
EDU+FE+HIS+F-UC	0.55	0.40	0.81	2.1	1.14	4.34
EDU+HV-UC	0.34	0.20	0.47	1.45	0.08	15.13
FE+UC	0.60	0.38	0.81	1.81	0.44	5.52

**Table 3:** Poison control center telephone number possession: Pooled effects median and HPD 95% credibility intervals.

treatments	Copula MONMA			Achana et al. MONMA (model 3)		
	median	2.50%	97.50%	median	2.50%	97.50%
EDU-UC	0.94	0.74	1.6	1.32	0.78	2.15
EDU+FE-UC	1.84	1.2	2.7	2.13	1.15	3.91
EDU+FE+HS-UC	1.36	0.82	2.33	1.95	1.12	3.93
EDU+FE+F-UC	0.66	0.37	1.67	1.26	0.67	2.59
EDU+HIS-UC	3.52	2.47	5.32	0.64	0.05	7.97
EDU+FE+HIS+F-UC	3.9	1.36	7.76	2.1	1.14	4.34
EDU+HV-UC	1.97	1.08	3	1.45	0.08	15.13
FE+UC	1.46	0.92	2.14	1.81	0.44	5.52

Table 4 reports median estimates and highest posterior density (HPD) credibility intervals for the between-study standard deviations and correlations. Again, the estimates produced by the fit of our model show a smaller variability especially in the estimation of the within-study correlations.

**Table 4:** Between-study standard deviations and correlations.

treatments	Copula MONMA			Achana et al. MONMA (model 3)		
	median	2.50%	97.50%	median	2.50%	97.50%
*σ*_1_	0.58	0.23	1.32	0.23	0.010	1.080
*σ*_2_	0.72	0.35	1.42	0.31	0.04	1.18
*σ*_3_	1.07	0.57	1.91	1.08	0.57	1.93
*ρ*^12^	0.02	-0.7	0.72	0.45	-0.99	1.00
*ρ*^13^	0.12	-0.75	0.83	0.5	-0.98	1.00
*ρ*^23^	0.40	-0.44	0.86	0.6	-0.87	0.99

### Alcohol dependence

In the second example, the data come from a Cochrane systematic review of pharmacology treatments for alcohol dependency. See [57, 58] for a detailed description of the methods and [59] for an update. The same data are also analysed in [43]. In particular, the authors model the outcome correlations resorting to a Clayton copula model, with one single parameter then fine-tuning such correlations. Moreover they allow for heterogeneity of the random effects. Again, our analysis can be replicated by running the script example_alcoholdependence.R to be found in the folder demo of the netcopula package.

**Fig 4:**
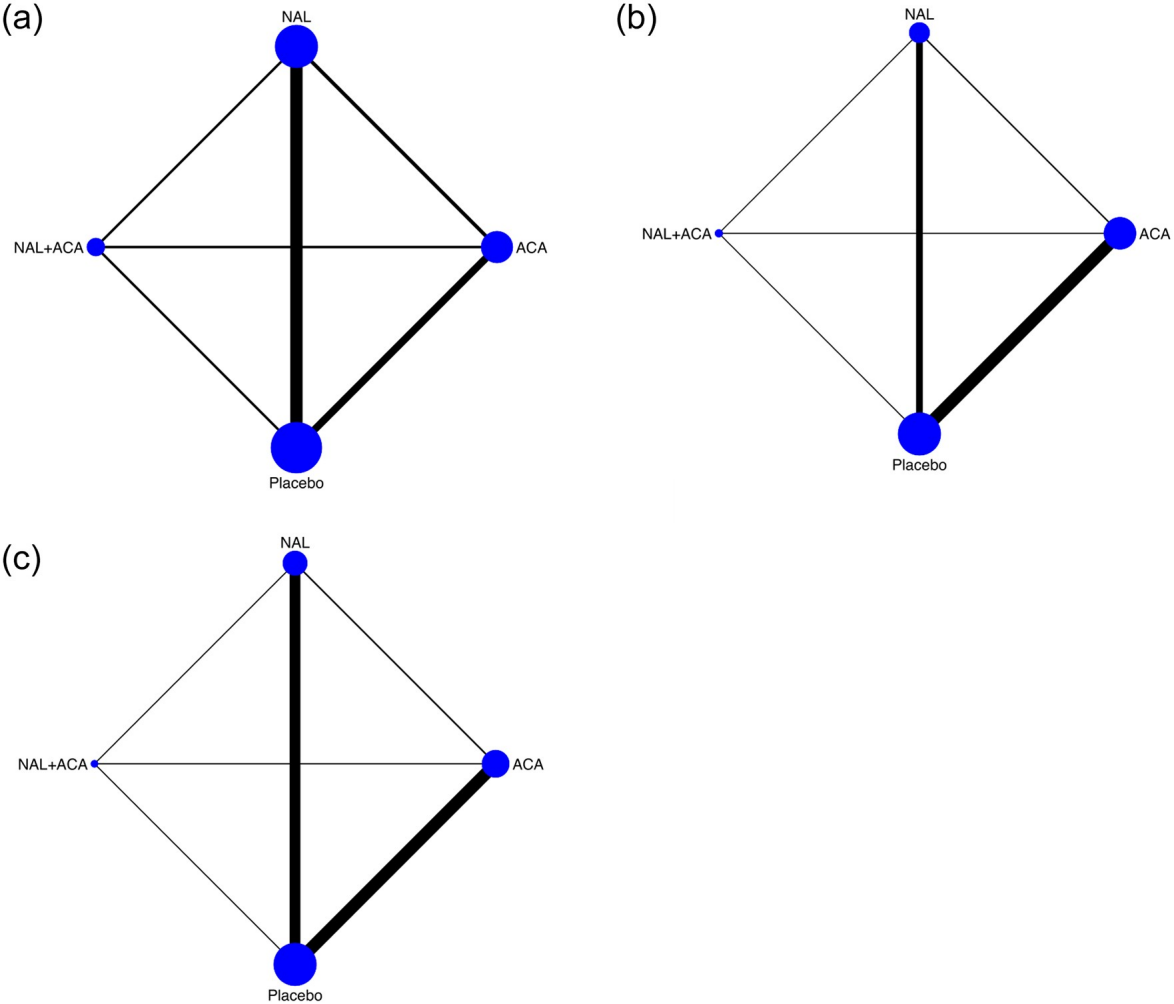
Alcohol dependence: The network structure. The figure shows the network structure: A: Return to Heavy Drinking, B: Return to Drinking and C: Discontinuation. The thicker the lines the higher the number of studies reporting results on the considered outcome.

The data come from forty-one studies and three outcomes are considered: Return to Heavy Drinking, Return to Drinking and Discontinuation. Eleven studies consider all three outcomes, twenty-two studies consider two outcomes and height report results only on one outcome. Three treatments are considered: naltrexone (NAL), acamprosate (ACA) and naltrexone + acamprosate (NAL+ACA). Fig 4 depicts the network graph for the three outcomes.

Tables 5 to 7 reports median estimates along HPD credibility intervals of the treatments pool effects obtained according to our model and [43] model. We can see that in all cases, our estimates show a smaller variability. Table 8 reports median estimates and credibility intervals for the within outcome correlations. Our model estimates a positive weak association between Return to Drinking and Discontinuation, even if there is high uncertainty on the estimation of the correlation between Return to Heavy Drinking and Return to Drinking and Return to Heavy Drinking and Discontinuation.

**Table 5:** Return to heavy drinking: Pooled effects posterior median and HPD 95% credibility intervals.

treatments	Copula MONMA			Liu et al. MONMA		
	median	2.50%	97.50%	median	2.50%	97.50%
NAL-PLB	0.51	0.35	0.69	0.47	0.33	0.62
ACA-PLB	0.73	0.44	1.01	0.68	0.47	0.97
NAL+ACA-PLB	0.5	0.26	0.86	0.5	0.31	0.74
ACA-NAL	1.4	0.89	2.08	1.47	0.99	2.25
NAL+ACA-NAL	0.99	0.53	1.7	1.08	0.64	1.65
NAL+ACA-ACA	0.71	0.39	1.25	0.75	0.43	1.11

**Table 6:** Return to drinking: Pooled effects posterior median and HPD 95% credibility intervals.

treatments	Copula MONMA			Liu et al. MONMA		
	median	2.50%	97.50%	median	2.50%	97.50%
NAL-PLB	0.61	0.44	0.83	0.57	0.41	0.75
ACA-PLB	0.51	0.42	0.59	0.52	0.41	0.65
NAL+ACA-PLB	0.4	0.26	0.6	0.4	0.22	0.64
ACA-NAL	0.83	0.59	1.15	0.94	0.65	1.29
NAL+AC-NAL	0.66	0.4	1.12	0.71	0.37	1.08
NAL+AC-ACA	0.78	0.52	1.25	0.76	0.44	1.25

**Table 7:** Discontinuation: Pooled effects posterior median and HPD 95% credibility intervals.

treatments	Copula MONMA			Liu et al. MONMA		
	median	2.50%	97.50%	median	2.50%	97.50%
NAL-PLB	0.78	0.63	0.97	0.75	0.55	0.99
ACA-PLB	0.8	0.65	0.97	0.81	0.66	1
NAL+ACA-PLB	0.75	0.42	1.28	0.82	0.47	1.4
ACA-NAL	1.03	0.78	1.32	1.1	0.79	1.49
NAL+ACA-NAL	0.96	0.54	1.58	1.1	0.59	1.75
NAL+AC-ACA	0.93	0.53	1.57	1.02	0.58	1.72

**Table 8:** Alcohol dependence: Within outcomes correlations.

correlations	Copula MONMA		
	median	2.50%	97.50%
Outcome 1—Outcome 2	0.62	-0.13	0.95
Outcome 1—Outcome 3	0.51	-0.23	0.92
Outcome 2—Outcome 3	0.78	0.25	0.96

Outcome 1 is Return to Heavy Drinking, Outcome 2 Return to Drinking and Outcome 3 is Discontinuation.

## Conclusion

In this paper we suggest a new model for a multiple outcomes network meta-analysis in the case of discrete outcomes. Our model accounts for both correlation between outcomes and between treatments. Moreover, we deal with the case of missing at random outcomes. In a comparison with two approaches previously proposed in the literature our results show a lower uncertainty. The model we suggest can be extended to the case of outcomes of different kinds, both discrete and continuous. We use here the Gaussian copula, but the model can be easily modified to include a different kind of copula.

However, there are some limitations in the suggested approach. The first problem arises in the estimation of the copula correlation matrix. The algorithm should be improved in order to reduce the running time and the uncertainty of the derived estimates. In the proposed approach the correlation matrix is assumed to be unstructured. This in the case of high-dimensional outcome might slow down the convergence of the algorithm. In this case a parametrization of the correlation matrix might be a reasonable choice as investigated in [60] and [61]. Moreover the performance of the model is particularly affected by the number of studies that in general is not high. More specifically, our model includes three orders of latent variables, the variables for the copula, the random effects and the latent variables to be introduced in the imputation step. The smaller number of studies considered in the first example (22) compared those in the second example (41) is in our opinions at the basis of the higher uncertainty in the estimation of the correlation parameters.

## Supporting information

S1 Appendix In the following we provide a description of the algorithm used for fitting the multiple outcome network meta-analysis model suggested. For the notation, we refer to the model depicted in Fig 1.
(PDF)
