# Supplementary material for: A Bayesian approach to discrete multiple outcome network meta-analysis
Source: PLoS One. 2020 Apr 28;15(4):e0231876. doi: 10.1371/journal.pone.0231876 (PMC7188248; doi:10.1371/journal.pone.0231876)
Supplement: S1 Appendix — For the notation, we refer to the model depicted in Fig 1. (PDF) [file pone.0231876.s001.pdf]

S1 Appendix. In the following we provide a description of the algorithm used for fitting the multiple outcome network meta-analysis model suggested. For the notation, we refer to the model depicted in Figure 1.

Likelihood

- After introducing the quantities $\boldsymbol{u}_{ik} = (u_{ik1}, \ldots, u_{ikM})^\top = (\Phi(x_{ik1}), \ldots, \Phi(x_{ikM}))^\top$, $i = 1, \ldots, n$ and $k \in \mathscr{T}_i$, we define the joint distribution of $(\boldsymbol{y}_{ik}, \boldsymbol{x}_{ik})$ as

$$f(\boldsymbol{y}_{ik}, \boldsymbol{x}_{ik}) = \prod_{m=1}^{M} \mathbb{I}\left(F_{ikm}(y_{ikm}^{-}) \leq \Phi(x_{ikm}) < F_{ikm}(y_{ikm})\right) c(\boldsymbol{u}_{ik}),$$

  where the indicator function $\mathbb{I}(A)$ equals 1 if A is true, and 0 otherwise, $F_{ikm}(\cdot)$ is the cumulative distribution function of a binomial random variable with parameters n_{ik} and p_{ikm}, and $c(\cdot)$ is the copula density of a Gaussian copula. The notation $F_{ikm}(y_{ikm}^{-})$ indicates the left limit of F_{ikm} at y_{ikm}, that is, $F_{ikm}(y_{ikm}^{-}) = F_{ikm}(y_{ikm} - 1)$.

- Since the parameters of the marginal distributions are the logit probabilities $\theta_{ikm} = \text{logit}(p_{ikm})$, $i = 1, \ldots, n$, $k \in \mathscr{T}_i$, $m = 1, \ldots, M$, while the parameters of

the copula functions are $\mathbf{\Gamma}_{\boldsymbol{k}}$, we can define the following augmented likelihood

$$\begin{aligned} f(\boldsymbol{y}, \boldsymbol{x} \mid \Theta, \mathbf{\Lambda}) &= \prod_{i=1}^{n} \prod_{k \in \mathscr{T}_i} f(\boldsymbol{y}_{ik}, \boldsymbol{x}_{ik} \mid \Theta_{ik}, \mathbf{\Gamma}_k) \\ &= \prod_{i=1}^{n} \prod_{k \in \mathscr{T}_i} c(\boldsymbol{u}_{ik}; \mathbf{\Gamma}_k) \prod_{m=1}^{M} \mathbb{I}\left(a_{ikm} \leq \Phi(x_{ikm}) < b_{ikm}\right), \end{aligned}$$

where $\boldsymbol{y} = \{\boldsymbol{y}_{ik} : i = 1, \ldots, n, k \in \mathscr{T}_i\}$, $\boldsymbol{x} = \{\boldsymbol{x}_{ik} : i = 1, \ldots, n, k \in \mathscr{T}_i\}$, $\boldsymbol{u} = \{\boldsymbol{u}_{ik} : i = 1, \ldots, n, k \in \mathscr{T}_i\}$, $\Theta = \{\Theta_{ik} : i = 1, \ldots, n, k \in \mathscr{T}_i\}$, with $\Theta_{ik} = \{\theta_{ik1}, \ldots, \theta_{ikM}\}$, $\mathbf{\Lambda} = \{\mathbf{\Gamma}_q : q \in \mathscr{T}\}$ $a_{ikm} = F_{ikm}(y_{ikm} - 1; \theta_{ikm})$ and $b_{ikm} = F_{ikm}(y_{ikm}; \theta_{ikm})$. Note that a_{ikm} and b_{ikm} are functions of both y_{ikm} and $\theta_{ikm} = \text{logit}(p_{ikm})$.

- Since $\theta_{ikm} = \text{logit}(p_{ikm})$ is a function of other parameters, namely μ_{im} and δ_{ikm}, the augmented likelihood can be rewritten as

$$\begin{aligned} f(\boldsymbol{y}, \boldsymbol{x} \mid \boldsymbol{\mu}, \boldsymbol{\delta}, \mathbf{\Lambda}) &= \prod_{i=1}^{n} \prod_{k \in \mathscr{T}_i} f(\boldsymbol{y}_{ik}, \boldsymbol{x}_{ik} \mid \boldsymbol{\mu}_i, \boldsymbol{\delta}_{ik}, \mathbf{\Gamma}_k) \\ &= \prod_{i=1}^{n} \prod_{k \in \mathscr{T}_i} c(\boldsymbol{u}_{ik}; \mathbf{\Gamma}_k) \prod_{m=1}^{M} \mathbb{I}\left(a_{ikm} \leq \Phi(x_{ikm}) < b_{ikm}\right) \end{aligned}$$

where $\boldsymbol{\mu} = \{\boldsymbol{\mu}_i : i = 1, \ldots, n\}$, $\boldsymbol{\delta} = \{\boldsymbol{\delta}_{ik} : i = 1, \ldots, n, k \in \mathscr{T}_i^-\}$, $a_{ikm} = F_{ikm}(y_{ikm}^-; \mu_{im}, \delta_{ikm})$ and $b_{ikm} = F_{ikm}(y_{ikm}; \mu_{im}, \delta_{ikm})$.

Posterior distribution

- The posterior distribution of the model is given by

$$\begin{aligned} \pi(\boldsymbol{\mu}, \boldsymbol{\delta}, \boldsymbol{d}, \mathbf{\Sigma}_M, \mathbf{\Lambda}, \boldsymbol{x} \mid \boldsymbol{y}) &\propto f(\boldsymbol{y}, \boldsymbol{x} \mid \boldsymbol{\mu}, \boldsymbol{\delta}, \mathbf{\Lambda}) \pi(\boldsymbol{\mu}) \pi(\boldsymbol{\delta} \mid \boldsymbol{d}, \mathbf{\Sigma}_M) \pi(\boldsymbol{d}) \pi(\mathbf{\Sigma}_M) \pi(\mathbf{\Lambda}) \\ &= \prod_{i=1}^{n} \prod_{k \in \mathscr{T}_i} c(\boldsymbol{u}_{ik}; \mathbf{\Gamma}_k) \prod_{m=1}^{M} \mathbb{I}\left(a_{ikm} \leq \Phi(x_{ikm}) < b_{ikm}\right) \\ &\times \prod_{i=1}^{n} \pi(\boldsymbol{\mu}_i) \prod_{i=1}^{n} \pi(\boldsymbol{\delta}_{i2}, \ldots, \boldsymbol{\delta}_{ia_i} \mid \boldsymbol{d}, \mathbf{\Sigma}_M) \pi(\boldsymbol{d}) \pi(\mathbf{\Sigma}_M) \\ &\times \prod_{q \in \mathscr{T}} \pi(\mathbf{\Gamma}_q) \end{aligned}$$

where $\boldsymbol{d} = \{\boldsymbol{d}_{r,q} : q \in \mathscr{T}\}$, with $r \in \mathscr{T}$.

Parameters update

The posterior distribution cannot be treated analytically, therefore posterior inferences are run through a Markov chain Monte Carlo (using a Gibbs-type updating of the parameters).

▷ *Copula parameters* $\boldsymbol{\Lambda}$

- At each iteration t we associate with $\boldsymbol{\Gamma}_q^{(t-1)}$ a covariance matrix $\boldsymbol{\Sigma}_q^{(t-1)}$ such that

$$\boldsymbol{\Gamma}_q^{(t-1)} = \left(\boldsymbol{D}_q^{(t-1)}\right)^{-1} \boldsymbol{\Sigma}_q^{(t-1)} \left(\boldsymbol{D}_q^{(t-1)}\right)^{-1}$$

  with $\boldsymbol{D}_q^{(t-1)} = \mathrm{diag}\left(\boldsymbol{\Sigma}_q^{(t-1)}\right)^{\frac{1}{2}}$ and $q \in \mathscr{T}$

- Then, we draw a proposal $\boldsymbol{\Sigma}_{q,prop}$ from an inverse Wishart distribution $\mathcal{IW}(\boldsymbol{S}_q, n_q)$, with

$$\boldsymbol{S}_q = \sum_{\{i:k=q\}} \left(\boldsymbol{D}_q^{(t-1)}\right)^{-1} \left(\boldsymbol{x}_{ik}^{(t-1)}\right) \left(\boldsymbol{x}_{ik}^{(t-1)}\right)^{\top} \left(\boldsymbol{D}_q^{(t-1)}\right)^{-1}, \tag{6}$$

  where the summation extends only to the n_q trials where treatment q is compared. Note that the number of degrees of freedom of the inverse Wishart is also equal to n_q.

- Finally, after working out the calculation

$$\boldsymbol{\Gamma}_{q,prop} = \mathrm{diag}(\boldsymbol{\Sigma}_{q,prop})^{-\frac{1}{2}} \boldsymbol{\Sigma}_{q,prop} \, \mathrm{diag}(\boldsymbol{\Sigma}_{q,prop})^{-\frac{1}{2}},$$

  at iteration t we accept the proposal $\boldsymbol{\Gamma}_{q,prop}$ using a Metropolis-Hastings step with acceptance probability

$$\alpha = \min\left\{1, \exp\left(\frac{M+1}{2}\left(\log|\boldsymbol{\Gamma}_{q,prop}| - \log|\boldsymbol{\Gamma}_q^{(t-1)}|\right)\right)\right\}.$$

- If one assumes $\boldsymbol{\Gamma}_q = \boldsymbol{\Gamma}$ for all $q \in \mathscr{T}$, the update needs to be modified by including all the n trials in Eq.(6). In this case we use a number of degrees of freedom equal to the (rounded) average number of arms per treatment.

▷ *Latent variables* $\boldsymbol{x}, \boldsymbol{\delta}$ *and* μ

- We propose to draw all these quantities simultaneously from the joint full conditional distribution

$$\pi(\boldsymbol{x}, \boldsymbol{\delta}, \boldsymbol{\mu} \,|\, \boldsymbol{d}, \boldsymbol{\Sigma}_M, \boldsymbol{\Lambda}, \boldsymbol{y})$$

- To sample from it we rely on a Gibbs sampler that loops through studies, treatments and components by drawing x_{ikm}, δ_{ikm} and μ_{im}, for all $i = 1, \ldots, n$, $k \in \mathscr{T}_i$ and $m = 1, \ldots, M$, from

$$\pi(x_{ikm}, \delta_{ikm}, \mu_{im} \,|\, \boldsymbol{x}_{i(-km)}, \boldsymbol{\delta}_{i(-km)}, \boldsymbol{\mu}_{i(-m)}, \boldsymbol{d}, \boldsymbol{\Sigma}_M, \boldsymbol{\Gamma}_k, y_{ikm}) =$$

$$\pi(\delta_{ikm}, \mu_{im} \,|\, \boldsymbol{x}_{i(-km)}, \boldsymbol{\delta}_{i(-km)}, \boldsymbol{\mu}_{i(-m)}, \boldsymbol{d}, \boldsymbol{\Sigma}_M, \boldsymbol{\Gamma}_k, y_{ikm}) \times \tag{7}$$

$$\pi(x_{ikm} \,|\, \boldsymbol{x}_{i(-km)}, \boldsymbol{\delta}_i, \boldsymbol{\mu}_i, \boldsymbol{\Gamma}_k, y_{ikm}) \tag{8}$$

where the notation $\boldsymbol{x}_{i(-km)}$ indicates all the elements of $\boldsymbol{x}_i$ (the set of latent variables related to study i) apart from the m-th component of its k-th arm, with a similar meaning for $\boldsymbol{\delta}_{i(-km)}$.

- To update these parameters we adopt the following strategy
- We first update δ_{ikm} and μ_{im} using a Gibbs-like approach, that is using their full conditionals from Eq.(7)

$$\pi(\delta_{ikm} \,|\, \boldsymbol{x}_{i(-km)}, \boldsymbol{\delta}_{i(-km)}, \boldsymbol{\mu}_i, \boldsymbol{d}, \boldsymbol{\Sigma}_M, \boldsymbol{\Gamma}_k, y_{ikm})$$

and

$$\pi(\mu_{im} \,|\, \boldsymbol{x}_{i(-km)}, \boldsymbol{\delta}_i, \boldsymbol{\mu}_{i(-m)}, \boldsymbol{d}, \boldsymbol{\Sigma}_M, \boldsymbol{\Gamma}_k, y_{ikm});$$

- then, we update x_{ikm} using Eq.(8).
- More specifically, these draws are performed as follows

- δ_{ikm} and μ_{im} are each updated by drawing respectively from

$$\pi(\delta_{ikm}\,|\,\boldsymbol{x}_{i(-km)}, \boldsymbol{\delta}_{i(-km)}, \boldsymbol{\mu}_i, \boldsymbol{d}, \boldsymbol{\Sigma}_M, \boldsymbol{\Gamma}_k, y_{ikm}) =$$

$$\pi(\delta_{ikm}\,|\,\boldsymbol{x}_{i(-km)}, \boldsymbol{\delta}_{i(-km)}, \mu_{im}, \boldsymbol{d}, \boldsymbol{\Sigma}_M, \boldsymbol{\Gamma}_k, y_{ikm}) \propto$$

$$\pi(y_{ikm}\,|\,\boldsymbol{x}_{i(-km)}, \delta_{ikm}, \mu_{im}, \boldsymbol{\Gamma}_k)\,\pi(\delta_{ikm}\,|\,\boldsymbol{\delta}_{i(-km)}, \boldsymbol{d}, \boldsymbol{\Sigma}_M)$$

$$= \left[\Phi\left(\frac{\Phi^{-1}(b_{ikm}) - \omega_{ikm}}{\gamma_{ikm}}\right) - \Phi\left(\frac{\Phi^{-1}(a_{ikm}) - \omega_{ikm}}{\gamma_{ikm}}\right)\right]\mathcal{N}(\delta_{ikm}; \tau_{ikm}, \eta^2_{ikm})$$

and

$$\pi(\mu_{im}\,|\,\boldsymbol{x}_{i(-km)}, \boldsymbol{\delta}_i, \boldsymbol{\mu}_{i(-m)}, \boldsymbol{d}, \boldsymbol{\Sigma}_M, \boldsymbol{\Gamma}_k, y_{ikm}) =$$

$$\pi(\mu_{im}\,|\,\boldsymbol{x}_{i(-km)}, \delta_{ikm}, \boldsymbol{\Gamma}_k, y_{ikm}) \propto \pi(y_{ikm}\,|\,\boldsymbol{x}_{i(-km)}, \delta_{ikm}, \mu_{im}, \boldsymbol{\Gamma}_k)\,\pi(\mu_{im})$$

$$= \left[\Phi\left(\frac{\Phi^{-1}(b_{ikm}) - \omega_{ikm}}{\gamma_{ikm}}\right) - \Phi\left(\frac{\Phi^{-1}(a_{ikm}) - \omega_{ikm}}{\gamma_{ikm}}\right)\right]\pi(\mu_{im})$$

with

- $a_{ikm} = F_{ikm}(y_{ikm} - 1; \mu_{im}, \delta_{ikm})$,
- $b_{ikm} = F_{ikm}(y_{ikm}; \mu_{im}, \delta_{ikm})$,
- $\omega_{ikm} = \boldsymbol{\Gamma}_{k,m,\backslash m}\left(\boldsymbol{\Gamma}_{k,\backslash m,\backslash m}\right)^{-1}\boldsymbol{x}_{i(-km)}$, and
- $\gamma^2_{ikm} = \boldsymbol{\Gamma}_{k,m,m} - \boldsymbol{\Gamma}_{k,m,\backslash m}\left(\boldsymbol{\Gamma}_{k,\backslash m,\backslash m}\right)^{-1}\boldsymbol{\Gamma}_{k,\backslash m,m}$,
- $\tau_{ikm} = d_{t_{i1},t_{ik},m} + \boldsymbol{\Sigma}_{M,m,\backslash m}\left(\boldsymbol{\Sigma}_{M,\backslash m,\backslash m}\right)^{-1}\left(\boldsymbol{\delta}_{i(-km)} - \boldsymbol{d}_{t_{i1},t_{ik},(-m)}\right)$,
- $\eta^2_{ikm} = \boldsymbol{\Sigma}_{M,m,m} - \boldsymbol{\Sigma}_{M,m,\backslash m}\left(\boldsymbol{\Sigma}_{M,\backslash m,\backslash m}\right)^{-1}\boldsymbol{\Sigma}_{M,\backslash m,m}$.

– We update each μ_{im} and δ_{ikm} using an adaptive random walk Metropolis-Hastings algorithm using a t proposal with $\nu = 7$ degrees of freedom as detailed below.

- x_{ikm} is updated (only for a non-missing y_{ikm}) by drawing from

$$\pi(x_{ikm}\,|\,\boldsymbol{x}_{i(-km)}, \boldsymbol{\delta}_i, \boldsymbol{\mu}_i, \boldsymbol{\Gamma}_k, y_{ikm}) =$$

$$= \pi(x_{ikm}\,|\,\boldsymbol{x}_{i(-km)}, \delta_{ikm}, \mu_{im}, \boldsymbol{\Gamma}_k, y_{ikm}) =$$

$$= \mathcal{N}(x_{ikm}; \omega_{ikm}, \gamma^2_{ikm})\,\mathbb{I}\left(a_{ikm} \le \Phi(x_{ikm}) < b_{ikm}\right)$$

We draw x_{ikm} from a univariate normal distribution which is truncated

between $\Phi^{-1}(b_{ikm})$ and $\Phi^{-1}(a_{ikm})$, where a_{ikm}, b_{ikm}, ω_{ikm} and γ^2_{ikm} are defined as above.

▷ *Pooled treatment effects across studies* $\boldsymbol{d}$ and *between-study covariance* $\boldsymbol{\Sigma}_M$

- We update $\boldsymbol{d}$ and $\boldsymbol{\Sigma}_M$ sequentially drawing from

$$\pi(\boldsymbol{d}, \boldsymbol{\Sigma}_M \mid \boldsymbol{x}, \boldsymbol{\mu}, \boldsymbol{\delta}, \boldsymbol{\Lambda}, \boldsymbol{y}) = \pi(\boldsymbol{d}, \boldsymbol{\Sigma}_M \mid \boldsymbol{\delta}) \propto \prod_{i=1}^{n} \pi(\boldsymbol{\delta}_{i2}, \ldots, \boldsymbol{\delta}_{ia_i} \mid \boldsymbol{d}, \boldsymbol{\Sigma}_M)\, \pi(\boldsymbol{d})\, \pi(\boldsymbol{\Sigma}_M) =$$

$$= \prod_{i=1}^{n} \mathcal{N}_M(\boldsymbol{\delta}_{i2}; \boldsymbol{d}_{t_{i1},t_{i2}}, \boldsymbol{\Sigma}_M) \prod_{k=3}^{a_i} \mathcal{N}_M\left(\boldsymbol{\delta}_{ik}; \boldsymbol{d}_{(k)}, \boldsymbol{\Sigma}_M^{(k)}\right) \pi(\boldsymbol{d})\, \pi(\boldsymbol{\Sigma}_M)$$

where

$$\boldsymbol{d}_{(k)} = \boldsymbol{d}_{t_{i1},t_{ik}} + \frac{1}{k-1} \sum_{j=2}^{k-1} (\boldsymbol{\delta}_{ij} - \boldsymbol{d}_{t_{i1},t_{ij}})$$

and

$$\boldsymbol{\Sigma}_M^{(k)} = \frac{k}{2(k-1)} \boldsymbol{\Sigma}_M.$$

In the previous calculation we used the standard approach in the network meta-analysis literature consisting in decomposing the joint density of $(\boldsymbol{\delta}_{i2}, \ldots, \boldsymbol{\delta}_{ia_i})$ into the corresponding marginal and conditional distributions for arm k, given the previous $1, \ldots, (k-1)$ arms.

- We first update $\boldsymbol{d}$ by drawing from

$$\begin{aligned}\pi(\boldsymbol{d} \mid \boldsymbol{\delta}, \boldsymbol{\Sigma}_M) &\propto \prod_{i=1}^{n} \pi(\boldsymbol{\delta}_{i2}, \ldots, \boldsymbol{\delta}_{ia_i} \mid \boldsymbol{d}, \boldsymbol{\Sigma}_M)\, \pi(\boldsymbol{d}) \\ &= \prod_{i=1}^{n} \mathcal{N}_M(\boldsymbol{\delta}_{i2}; \boldsymbol{d}_{t_{i1},t_{i2}}, \boldsymbol{\Sigma}_M) \prod_{k=3}^{a_i} \mathcal{N}_M\left(\boldsymbol{\delta}_{ik}; \boldsymbol{d}_{(k)}, \boldsymbol{\Sigma}_M^{(k)}\right) \pi(\boldsymbol{d}),\end{aligned}$$

where we used the same notation as above. We update $\boldsymbol{d}$ using an adaptive random walk Metropolis-Hastings algorithm with a multivariate t proposal with $\nu = 7$ degrees of freedom, which is centered around the previous iteration value $\boldsymbol{d}^{(t-1)}$, and with scale matrix $\kappa_{\boldsymbol{d}} \boldsymbol{\Psi}^{(t)}$ with $\kappa_{\boldsymbol{d}} = (2.38^2/M)$ and where $\boldsymbol{\Psi}^{(t)}$ is updated every $T = 100$ iterations as follows

  - at iteration $t = 0$, set starting values $\boldsymbol{\upsilon}^{(0)} = \boldsymbol{0}$ and $\boldsymbol{\Psi}^{(0)} = \boldsymbol{I}$,

- at iteration $t = b\,T$, with $b = 1, 2, 3, \ldots$, update the proposal distribution variance $\boldsymbol{\Psi}^{(t)}$ with the following two steps

$$\boldsymbol{v}^{(b)} = \boldsymbol{v}^{(b-1)} + \frac{1}{T} \sum_{j=(b-1)\,T+1}^{b\,T} \left(\boldsymbol{d}^{(j)} - \boldsymbol{v}^{(b-1)}\right)$$

$$\boldsymbol{\Psi}^{(b)} = \boldsymbol{\Psi}^{(b-1)} + \frac{1}{T} \sum_{j=(b-1)\,T+1}^{b\,T} \left[\left(\boldsymbol{d}^{(j)} - \boldsymbol{v}^{(b-1)}\right)\left(\boldsymbol{d}^{(j)} - \boldsymbol{v}^{(b-1)}\right)^{\top} - \boldsymbol{\Psi}^{(b-1)}\right].$$

– To draw $\boldsymbol{d}$, we stack the $\boldsymbol{d}_{r,s}$ values and draw them jointly from a multivariate t with size $M \times \left(\dim(\mathscr{T}) - 1\right)$. The corresponding scale matrix will be block diagonal reflecting the fact that the different pooled treatment effects $\boldsymbol{d}_{r,s}$ are assumed to be a priori independent.

– Reminding that $\boldsymbol{\Sigma}_M$ has a prior distribution parameterized in terms of a log-Cholesky factorization of its inverse with elements assumed to be a priori independent and distributed according to a normal distribution with zero mean and variance σ_r^2, its update is undertaken by generating the elements β_ℓ of the log-Cholesky parameterization

$$\pi(\beta_\ell \,|\, \{\boldsymbol{\beta} \backslash \beta_\ell\}, \boldsymbol{\delta}, \boldsymbol{d}) \propto \prod_{i=1}^{n} \pi(\boldsymbol{\delta}_{i2}, \ldots, \boldsymbol{\delta}_{ia_i} \,|\, \boldsymbol{d}, \boldsymbol{\Sigma}_M)\, \pi(\beta_\ell) =$$

$$= \prod_{i=1}^{n} \mathcal{N}_M(\boldsymbol{\delta}_{i2}; \boldsymbol{d}_{t_{i1},t_{i2}}, \boldsymbol{\Sigma}_M) \prod_{k=3}^{a_i} \mathcal{N}_M\left(\boldsymbol{\delta}_{ik}; \boldsymbol{d}_{(k)}, \boldsymbol{\Sigma}_M^{(k)}\right) \mathcal{N}(\beta_\ell; 0, \sigma_r^2)$$

using an adaptive random-walk Metropolis-Hastings algorithm with an adaptation similar to that described for $\boldsymbol{d}$. Once an iterate of $\boldsymbol{\beta}$ is obtained, the iterate of $\boldsymbol{\Sigma}_M$ can be computed using the relationship $\boldsymbol{\Sigma}_M = \boldsymbol{R}^{-1}\left(\boldsymbol{R}^{-1}\right)^{\top}$. As for the proposal distribution of the β_ℓ, we use a Cauchy distribution centered around zero and with scale $\kappa_r \zeta^2$, where we choose $\kappa_r = 2.38^2$. The proposal parameter ζ^2 is initialized at 1 and then tuned during the simulation according to the adaptation schedule.

Missing data

- We impute the missing data as part of the estimation process. In particular, we need to impute only the latent variables $\boldsymbol{x}$ associated with the missing outcomes.

- We denote with $\boldsymbol{x}_{ik,obs}$ the vector of latent variables associated with the observed outcomes for study i and treatment $k \in \mathscr{T}_i$, and with $\boldsymbol{x}_{ik,miss}$ the corresponding vector associated with the missing outcomes.

- At the beginning of each iteration we jointly impute the missing values of *all* the latent variables related to a common treatment q. We start by collecting the latent variables related to a common treatment q, that is $\boldsymbol{x}_{(q)} = (\boldsymbol{x}_{1q}, \ldots, \boldsymbol{x}_{n_q q})$, with n_q denoting the number of trials where treatment q is compared, which are jointly distributed as

$$f\left(\boldsymbol{x}_{(q)} \mid \boldsymbol{\Gamma}_q\right) = \mathcal{N}_{M \cdot n_q}\left(\boldsymbol{0}, \widetilde{\boldsymbol{\Gamma}}_q\right),$$

where $\widetilde{\boldsymbol{\Gamma}}_q$ denotes the block-diagonal correlation matrix

$$\widetilde{\boldsymbol{\Gamma}}_q = \boldsymbol{I}_{n_q} \otimes \boldsymbol{\Gamma}_q = \begin{pmatrix} \boldsymbol{\Gamma}_q & \boldsymbol{O} & \cdots & \boldsymbol{O} \\ & \boldsymbol{\Gamma}_q & \cdots & \boldsymbol{O} \\ & & \ddots & \vdots \\ & & & \boldsymbol{\Gamma}_q \end{pmatrix},$$

with "$\otimes$" denoting the Kronecker product of two matrices, $\boldsymbol{O}$ representing the null $(M \times M)$ matrix and $\boldsymbol{\Gamma}_q$ indicating the $(M \times M)$ arm-specific correlation matrix relative to treatment $q \in \mathscr{T}_i$.

- We then partition the elements of $\boldsymbol{x}_{(q)}$ as $(\boldsymbol{x}_{(q),miss}, \boldsymbol{x}_{(q),obs})$, such that at iteration t we impute all the missing values $\boldsymbol{x}_{(q),miss}$ by drawing from the distribution

$$f\left(\boldsymbol{x}^{(t)}_{(q),miss} \mid \boldsymbol{x}_{(q),obs}, \boldsymbol{\Gamma}^{(t-1)}_q\right) \quad = \quad \mathcal{N}_{\sum_{i=1}^{n_q} h_i}\left(\widetilde{\boldsymbol{\mu}}_{q,miss \mid obs}, \widetilde{\boldsymbol{\Omega}}^{(t-1)}_{q,miss \mid obs}\right),$$

where h_i represents the number of missing elements in $\boldsymbol{x}^{(t)}_{iq,miss}$ for the i-th study where treatment q is compared,

$$\widetilde{\boldsymbol{\mu}}_{q,miss \mid obs} = \widetilde{\boldsymbol{\Gamma}}^{(t-1)}_{q,12}\left(\widetilde{\boldsymbol{\Gamma}}^{(t-1)}_{q,obs}\right)^{-1} \boldsymbol{x}_{(q),obs},$$

$$\widetilde{\boldsymbol{\Omega}}^{(t-1)}_{q,miss \mid obs} = \widetilde{\boldsymbol{\Gamma}}^{(t-1)}_{q,miss} - \widetilde{\boldsymbol{\Gamma}}^{(t-1)}_{q,12}\left(\widetilde{\boldsymbol{\Gamma}}^{(t-1)}_{q,obs}\right)^{-1} \widetilde{\boldsymbol{\Gamma}}^{(t-1)}_{q,21},$$

and the sub-matrices of $\widetilde{\mathbf{\Gamma}}_q^{(t-1)}$ are taken from the following decomposition

$$\widetilde{\mathbf{\Gamma}}_{q,reshaped}^{(t-1)} = \begin{pmatrix} \widetilde{\mathbf{\Gamma}}_{q,miss}^{(t-1)} & \widetilde{\mathbf{\Gamma}}_{q,12}^{(t-1)} \\ \widetilde{\mathbf{\Gamma}}_{q,21}^{(t-1)} & \widetilde{\mathbf{\Gamma}}_{q,obs}^{(t-1)} \end{pmatrix}$$

after its rows and columns have been reordered accordingly.
